# Application of an Innovative Methodology to Build Infrastructure for Digital Transformation of Health Systems: Developmental Program Evaluation

**DOI:** 10.2196/53339

**Published:** 2025-04-17

**Authors:** M Claire Buchan, Tarun Reddy Katapally, Jasmin Bhawra

**Affiliations:** 1 School of Public Health Sciences University of Waterloo Waterloo, ON Canada; 2 DEPtH Lab, Faculty of Health Sciences Western University London, ON Canada; 3 Department of Epidemiology and Biostatistics Schulich School of Medicine and Dentistry Western University London, ON Canada; 4 Lawson Health Research Institute London, ON Canada; 5 CHANGE Research Lab, School of Occupational and Public Health Toronto Metropolitan University Toronto, ON Canada

**Keywords:** digital health platform, citizen science, evaluation, health systems, digital health, app, innovative, digital transformation, public health, crises, communicable disease, coronavirus, chronic diseases, decision-making, assessment, thematic analysis, self-report survey, risk, artificial intelligence, AI

## Abstract

**Background:**

The current public health crises we face, including communicable disease pandemics such as COVID-19, require cohesive societal efforts to address decision-making gaps in our health systems. Digital health platforms that leverage big data ethically from citizens can transform health systems by enabling real-time data collection, communication, and rapid responses. However, the lack of standardized and evidence-based methods to develop and implement digital health platforms currently limits their application.

**Objective:**

This study aims to apply mixed evaluation methods to assess the development of a rapid response COVID-19 digital health platform before public launch by engaging with the development and research team, which consists of interdisciplinary researchers (ie, key stakeholders).

**Methods:**

Using a developmental evaluation approach, this study conducted (1) a qualitative survey assessing digital health platform objectives, modifications, and challenges administered to 5 key members of the software development team and (2) a role-play pilot with 7 key stakeholders who simulated 8 real-world users, followed by a self-report survey, to evaluate the utility of the digital health platform for each of its objectives. Survey data were analyzed using an inductive thematic analysis approach. Postpilot test survey data were aggregated and synthesized by participant role.

**Results:**

The digital health platform met original objectives and was expanded to accommodate the evolving needs of potential users and COVID-19 pandemic regulations. Key challenges noted by the development team included navigating changing government policies and supporting the data sovereignty of platform users. Strong team cohesion and problem-solving were essential in the overall success of program development. During the pilot test, participants reported positive experiences interacting with the platform and found its features relatively easy to use. Users in the community member role felt that the platform accurately reflected their risk of contracting COVID-19, but reported some challenges interacting with the interface. Those in the decision maker role found the data visualizations helpful for understanding complex information. Both participant groups highlighted the utility of a tutorial for future users.

**Conclusions:**

Evaluation of the digital health platform development process informed our decisions to integrate the research team more cohesively with the development team, a practice that is currently uncommon given the use of external technology vendors in health research. In the short term, the developmental evaluation resulted in shorter sprints, and the role-play exercise enabled improvements to the log-in process and user interface ahead of public deployment. In the long term, this exercise informed the decision to include a data scientist as part of both teams going forward to liaise with researchers throughout the development process. More interdisciplinarity was also integrated into the research process by providing health system training to computer programmers, a key factor in human-centered artificial intelligence development.

## Introduction

### Background

The current public health crises we face are global in nature, ranging from communicable disease pandemics, such as COVID-19, to endemic noncommunicable diseases and long-term care burdens [1,2]. The presence of multiple overlapping health issues, termed a *syndemic*, is further complicated in the age of polycrises. Crisis responses increasingly require coordination from multiple sectors, including, but not limited to, health care, environment, and social services [3,4]. In order to address the gaps in our current systems of care, deliberate and cohesive societal efforts are required to understand and respond to existing inefficiencies in health systems.

Digital health platforms, which range from mobile health apps and virtual care products to digital health dashboards [5,6], have immense potential to transform our health systems by increasing access to care, predicting symptoms and outcomes, and enabling rapid responses to health crises [4,6-9]. For instance, digital health platforms can enable patients to connect with their health care providers remotely [10,11], and, in turn, health care providers are able to predict risks by ethically leveraging big data from patients [12,13] and provide support by engaging with patients remotely [10,14]. More importantly, the application of digital health platforms is not limited to patients; that is, they can be used by apparently healthy individuals to self-monitor and track their health behaviors and outcomes [15,16] as well as share their data ethically with health care providers and scientists [13,17]. Given the ubiquity of digital devices [18-20], their adaptability, and their reach across geographic regions and sociodemographic groups, digital health platforms are capable of bridging existing gaps in health information and care access [6,21,22]. More importantly, such platforms, particularly with the incorporation of artificial intelligence (AI) and machine learning, can enable precision prediction of health outcomes [23,24] as well as rapid responses to help monitor, mitigate, and manage existing and emerging health crises [6,25]. While the development of digital health platforms has increased significantly in the past decade, and in particular during the COVID-19 pandemic [26], there are no standardized processes for development and evaluation to ensure that evidence-based approaches are applied using interdisciplinary expertise.

The role of digital health platforms in managing public health and promoting health care access is predicted to grow exponentially [27]. Thus, it is critical that the development of these platforms is evaluated using rigorous methods to ensure effectiveness and efficacy. The World Health Organization recently released a guide for evaluating digital health interventions [28], and many other groups have adapted methods to assess digital platform technology [29-31]. However, these guidelines do not provide directions to evaluate digital platform *development processes*, including prototype development sprints and troubleshooting [28], which are key steps that come well ahead of the actual implementation of digital interventions.

Another key challenge is ensuring data privacy and data sovereignty of digital health platforms, particularly when serving communities that have been historically disenfranchised or discriminated against. Data sovereignty refers to meaningful control or ownership of one’s own data and is a critical aspect of self-governance and self-determination, particularly among Indigenous and other colonized communities [32,33]. In creating digital health platforms that serve these communities, the development process thus requires the integration of rigid privacy and data protocols, in addition to consideration of culturally appropriate features tailored to communities’ specific needs [4,7,32,34]. Moreover, as technology continues to evolve, it is crucial to evaluate the development and use of AI, especially to ensure that AI is designed in a way that is truly human centered [35,36]. Evaluating development processes helps to identify and mitigate the risks associated with AI, which could include neglecting user needs, overlooking the social impact, and failing to address biases and accountability issues [37]. By systematically evaluating development processes, we can foster AI systems that are better aligned with human values and needs (ie, health services) [36].

### Study Objective

Overall, the evaluation of digital health platform development has enormous consequences for eventual citizen health and well-being, as well as data safety, security, and data sovereignty [38]. The potential for causing harm to populations, the sensitivity of personally identifiable big data that are collected via digital health platforms, as well as the need to develop human-centered AI require a rigorous evaluation process, including internal pilot testing before public testing. Most importantly, a significant gap exists in the peer-reviewed literature regarding evaluation approaches for the development of evidence-based digital health platforms, particularly in academic settings, where there is a critical need for co-design [39] not only from a citizen or patient perspective, but also by cohesive engagement between research and development teams. To address this critical gap in digital health platform development, this study aimed to apply mixed evaluation methods to assess the development of a digital health platform that was exclusively developed by a research and development team working remotely during the COVID-19 pandemic to manage, monitor, and mitigate household risk of COVID-19.

## Methods

### CO-Away Digital Health Platform

**Figure 1:**
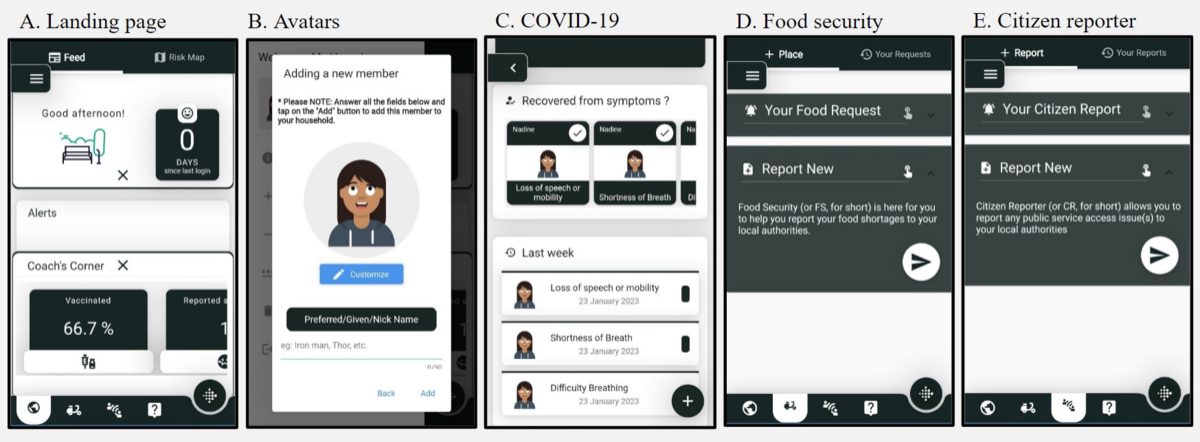
Screenshots of the CO-Away platform interface for community members, including the platform landing page, avatar creation process, COVID-19 symptom assessment, food security request, and citizen reporter feature.

In an effort to address the imminent public health crisis of the COVID-19 pandemic, the CO-Away digital health platform was developed to track, manage, and mitigate household risk of COVID-19. During the pandemic, rural, remote, and Northern communities in Canada were disproportionately impacted and experienced challenges with health information, data sovereignty, and care access [40]. In the Canadian context, Indigenous communities reported gaps in access to health information and care [34,41]; thus, after conducting a comprehensive needs assessment [34], the CO-Away platform was developed to enable near real-time monitoring, rapid response, and health care data access. CO-Away comprises a progressive web application for users to manage household COVID-19 risk as well as a backend digital decision-making dashboard that visualizes aggregated and anonymized big data relayed in real time from the progressive web application [6]. Progressive web applications are a type of web application that leverage web technologies to provide a more app-like user experience, combining the best features of web applications and mobile apps [42]. Figure 1 shows screenshots of the CO-Away home page and dashboard. The CO-Away platform provides local jurisdictional decision makers access to aggregate-level data to track and respond to emerging risk patterns and trends in near real time.

Given the wide-ranging impacts of the COVID-19 pandemic on other aspects of health and wellness, CO-Away takes a holistic approach that extends beyond COVID-19 symptom assessment to include other key digital platform features, such as (1) food security request, which helps monitor and manage food shortages within a jurisdiction and (2) citizen reporter, which reports any public service or access issues experienced by community members [6]. CO-Away serves as a link to connect citizens with decision makers and offers value to both households and decision makers. This digital health platform, driven by citizen-generated big data collected from ubiquitous tools, not only supports citizens by providing them with real-time support and valuable insights to enhance their decision-making abilities, but also aggregates and anonymizes citizen data. These aggregated, anonymized data are then delivered to decision makers, enabling real-time exchanges of information and alerts through direct bidirectional engagement between citizens and decision makers. This innovative approach represents a paradigm shift in the way community health is approached, placing priority on addressing the immediate needs of citizens. This study evaluated the development processes of both the app that citizens or community members use and the digital health dashboard that decision makers use. Additional details on the CO-Away platform can be found in another publication [43].

### Evaluation Approach

To examine the evolution, challenges, successes, and utility of the CO-Away platform, a mixed methods developmental evaluation was performed after the completion of the first prototype to inform subsequent iterations of the platform. Developmental evaluations generate learnings to inform the development of an initiative; thus, are often used in complex and unpredictable scenarios, such as the COVID-19 pandemic [44,45]. A developmental evaluation is utilization focused and should be designed and implemented in ways that maximize utility for the primary intended users [46]. This evaluation approach generates data and findings in near real time, thereby facilitating developmental decision-making and course corrections throughout the development process [44,47].

### Evaluation Questions

This evaluation was guided by the following overarching evaluation questions:

- What factors influenced the CO-Away digital health platform development?
- How has the CO-Away digital platform development evolved over the course of the project?
- To what extent does the CO-Away digital health platform achieve its goals and objectives from the perspective of community members or users and decision makers?
- How do these findings translate to the development process?
- What direction will the CO-Away project take going forward?

### Evaluation Design

The evaluation included the following two key components: (1) a qualitative survey assessing program objectives, modifications, and challenges administered to 5 key members of the software development team and (2) a role-playing pilot test conducted with key stakeholders who were part of the research team, followed by a self-report survey, to evaluate the utility of the program for each of its goals or objectives.

### Data Collection

Data collection for the CO-Away developmental evaluation included two primary components: (1) a development team survey (refer to Multimedia Appendix 1 for the survey) and (2) a research team pilot test.

#### Part A: Development Team Survey

A total of 5 members of the CO-Away software development team were asked to complete a web-based self-report survey. The survey was designed to assess the evolution of program objectives, target audience, and key modifications made throughout the development process. A guiding framework developed for conducting needs assessments to conceptualize and implement digital infrastructure was used to guide the development of this evaluation survey [34]. These key team members were identified by the principal investigator and the evaluation team and included software developers and data scientists. Participants were all involved in the development of CO-Away and project organization or management. Due to the potential for bias that could arise from having key members of the development team involved in the data collection process, participants were not involved in the preparation of the evaluation survey and were blinded to the responses of their fellow interviewees.

Participants were asked to complete the survey within a 1-week period from July 18, 2022, to July 25, 2022. The survey was designed to be completed in <60 minutes and was administered through Qualtrics (Qualtrics International Inc) [48]. Participants were asked to describe their role in the digital platform design, any major deviations from the initial project proposal, any changes in the target audience, and the digital platform’s potential for impact using open text fields. Participants were also asked to reflect on the barriers to and facilitators of developing the CO-Away platform within the context of the COVID-19 pandemic.

#### Part B: Pilot Test

**Figure 2:**
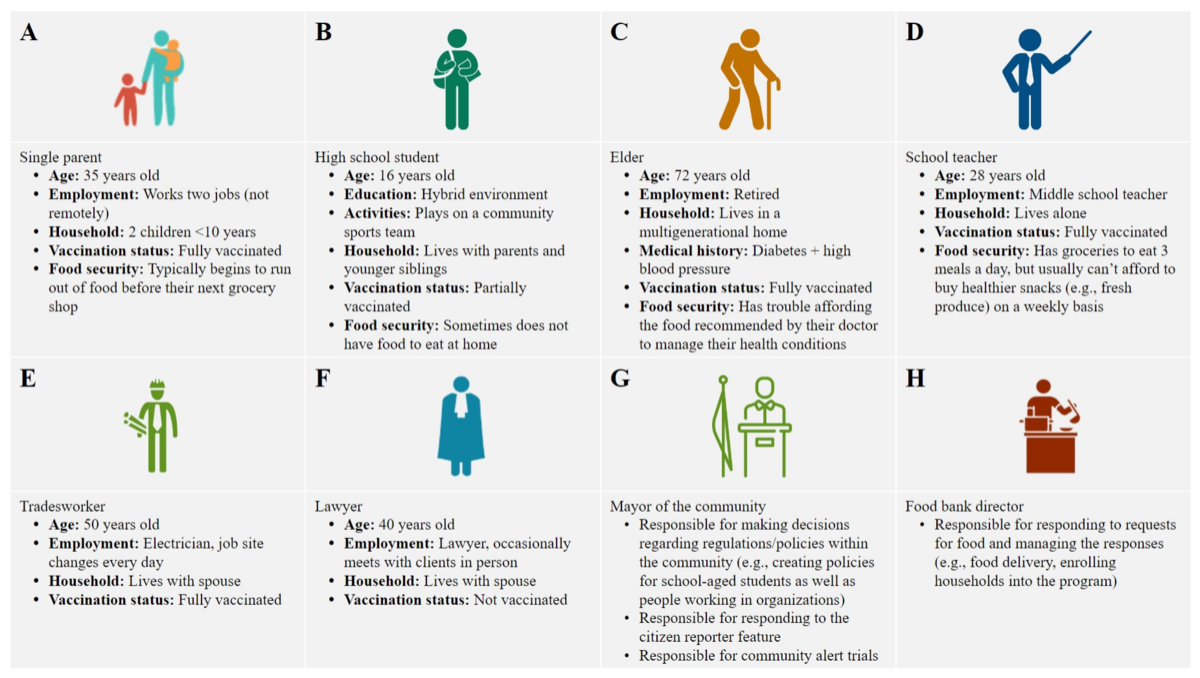
Overview of the 8 pilot test character descriptions.

To evaluate the utility of the CO-Away platform for each of its objectives, a role-playing pilot test was conducted with 7 key stakeholders who were part of the interdisciplinary research team. All project stakeholders had expertise in digital health, epidemiology, and public health. Project stakeholders assumed the role of 1 of the 8 character roles developed by the evaluation team. These character roles consisted of 6 (75%) community members and 2 (25%) decision makers. One project stakeholder was asked to assume 2 roles within the pilot test, both of a community member and a decision maker, to reflect a real-world scenario where decision makers can be community members requiring access to both digital dashboards, that is, dual roles. The evaluation team developed the characters to simulate a range of potential user backgrounds and provided each participant with a brief overview of their character detailing their age, household composition, occupation, remote work ability, vaccination status, and food security status (Figure 2). Participating stakeholders reviewed the list of character descriptions and submitted their preferred roles to the evaluation team.

Participants were assigned their character role for the duration of the pilot test and were asked to use their provided character descriptions to create avatars within the CO-Away app. Participants were advised to use their assigned character description as a starting point and to interact with the platform as they saw fit to simulate real-world conditions. Participants were instructed to improvise their engagement with specific features of the platform (eg, COVID-19 diagnoses and symptoms). The participant who assumed both a decision maker and community member role set up 2 distinct profiles and was asked to engage with the platform (to the best of their ability) under each role independently. The pilot test was conducted over a 2-week period from July 8, 2022, to July 21, 2022. Over the course of the activity, participants were sent daily reminders via email to input their data into the app using the integrated features.

Following the pilot test, participants were asked to complete a brief self-report survey detailing their experience using the app by giving them a variety of response options, including yes or no and Likert scale questions. The survey assessed the general usability of the app and specific app features, including COVID-19 risk, food security, and citizen reporter features. Two surveys were developed by the evaluation team, one for community member participants (Multimedia Appendix 2) and the other for the decision maker participants **(**Multimedia Appendix 3). The survey administered was dependent on the participants’ assumed roles in the study (ie, community members or decision makers), while some general questions were asked of both groups, and most questions differed between groups. The one participant who assumed both a community member and a decision maker role was asked to complete both surveys. The survey was designed to be completed in 30 minutes and was distributed electronically using Qualtrics [48].

By accessing the cloud-based database [6], daily user engagement reports were generated over the course of the pilot test to examine user engagement across the features of the platform. Reports included data on user engagement (eg, the number of new sign-ups and total log-ins), platform usability (eg, the number of households reporting their behaviors, the number of food requests, and the number of food requests processed and delivered), and reported issues (eg, the number of reported issues using the platform).

### Ethical Considerations

The research and development team who participated in this project either provided simulated data as part of a role play scenario, or were subject matter experts, respectively. This developmental evaluation enabled testing of the functionality, usability, and areas of improvement for the digital platform, thus standard research ethics requirements did not apply [48]. However, survey response data remained anonymous to protect participant privacy, and survey responses were not associated with individual software development team members. Moreover, all data collected in the role-play exercise were simulated hypothetical scenarios, did not reflect true participant behaviors, and thus did not require strict anonymity and privacy protocols. Participants were not compensated for their participation in this evaluation.

### Analysis

Data from the development team survey were downloaded from Qualtrics and synthesized in Microsoft Word. Data were analyzed using an inductive thematic analysis approach. To generate an initial coding manual, 1 evaluation team member (MCB) independently reviewed the first survey and organized the data into themes and meaningful codes. A second evaluation team member (JB) subsequently reviewed the coded transcript to ensure consistency and agreement. Once all transcripts were coded by 1 evaluation team member (MCB), the evaluation team (MCB and JB) reconvened to discuss relationships between codes that could be grouped into themes.

Data from the postpilot test survey were downloaded from Qualtrics and aggregated in Microsoft Excel. Data were analyzed and summarized for each question and results were grouped by community members and decision makers. Result tables were sorted according to question topic areas (eg, COVID-19, food security, and citizen reporter). Quotations in the result tables indicated participant responses to open text fields on the survey. For questions where participants could select more than 1 option, responses were totaled across categories, and, as such, percentages may add to more than 100.

## Results

### Part A: Development Team Survey

#### Overview

All 5 members of the software development team completed the survey. The roles of the participating team members included the principal investigator (n=1, 20%), 3 (60%) software developers—one of whom was involved in project organization and management—and 1 (20%) project coordinator. The 5 subsequent subsections describe the key themes that arose through analysis of the development survey.

#### Theme 1: Alignment With Original Objectives

Over the course of the development process, several modifications to the app structure were made, many of which were made to accommodate the evolving nature of the COVID-19 pandemic. These changes included modifying existing features of the app (ie. the COVID-19 feature) and the addition of new features (eg, the food security feature and citizen reporter). Despite these modifications, the overall objective of CO-Away remained consistent throughout development. One participant noted the following:

The prototype satisfies the originally planned platform’s mandate. The changes that took place were more logistical, which were beyond the scope of the development team. The critical takeaway is that the concept and the ultimate impact remain the same.

DT5

In addition, the team noted improvements in the visual appearance of the app. Overall, the development team indicated that the final app not only met the original objectives but also the plan expanded to accommodate the evolving needs of the community and COVID-19 regulations.

#### Theme 2: Project Challenges

There were a series of challenges identified by the development team, including tailoring the platform to a diverse audience (ie, both young and older community members), learning new software development tools (eg, programming languages), and the strict regulations surrounding COVID-19–related platforms on mobile app stores. The CO-Away app required integrating various disciplines to address community health needs, which was described as a unique challenge:

As this project’s goal is to evaluate a COVID-19 mitigating digital health platform for Indigenous self-governance, determination, and data sovereignty in a remote [Indigenous] community...the initiative from its inception has been complex not only due to the integration of multiple disciplines [epidemiology, data science, Indigenous health, public health, among others], but also due to the ultimate purpose of the digital platform: rapidly responding to community needs.

DT5

A digital platform developed for a remote Indigenous community app would have a substantially greater chance for success if it took a holistic approach, reiterating Traditional Indigenous Knowledge of holistic health. This approach required continuous collaboration with the citizen scientist advisory council [6,34], and a part of this process required developing strategies and features within the app that manage and mitigate other population health crises that were worsened by the COVID-19 pandemic (eg, food security, mental health and substance misuse, and negative interactions of youth with law enforcement).

One of the key challenges that was pertinent for COVID-19 app development, in particular, was constantly evolving government policies. Participants reported that several previously existing features required repeated modifications, and features not initially outlined in the project plan required development on the go. For example, one participant commented that calculating their risk of COVID-19 required incorporating evolving guidelines on vaccination dosage:

...changes with increase in the number of doses of vaccination.

DT4

The nature of the COVID-19 pandemic not only required the software development team to adapt to changing government policies but also to the evolution of the virus variants themselves. Another participant highlighted the addition of the vaccine passport feature to the app, which was not within the original project scope.

While the ability of team members to work remotely was noted as a facilitator of project execution, participants underscored the delays that resulted from the constant need to update program features to align with government policies. Of high importance were the challenges associated with supporting the data sovereignty of the platform users. One participant commented the following:

We have had to go above and beyond existing data safety regulations in the world to build a digital health platform that provides Indigenous citizens control and ownership over their data.

DT5

Despite the plethora of challenges posed by the COVID-19 pandemic, the software development team was able to adapt their approach and practices to build a successful app.

#### Theme 3: Factors Influencing App Development

There was a range of factors that influenced app development, particularly for an app focused on addressing the rapidly changing virus during the COVID-19 pandemic. In particular, participants noted that working during the COVID-19 pandemic posed specific barriers to the recruitment of developers. Moreover, navigating the development process dominated by, and dependent on big technology companies (Big Tech), as well as a shift to a new academic institution for the principal investigator were key external factors that influenced overall app development. Recruitment challenges and a change in academic institution created logistical challenges that added to the complexity of this large-scale project:

Due to the restriction of movement during the pandemic, we faced significant difficulties to recruit high-quality personnel. This issue is especially challenging in the disciplines of computer science and data science, where most of the personnel who fit our criteria are recent international graduates, who face IRCC [Immigration, Refugees and Citizenship Canada] backlogs for work permits.

DT5

One challenge noted by several participants was launching a platform in Big Tech app stores, which ultimately resulted in the creation of a progressive web application. For example, one participant stated the following:

To truly ensure self-determination and data sovereignty, we had to abandon launching our digital health platform on Apple Store and Google PlayStore to eliminate the control of these stores over the development process. This decision meant the development of a progressive web-based platform that does not need these stores to be launched and the platform would only be provided to the community members via a password protected process.

DT5

The 2 primary app hosting Big Tech companies, Apple Store and Google Play Store, implemented strict regulations around launching COVID-19–related platforms on their mobile app stores, which hindered the development process. While this transition created delays and roadblocks that the development team needed to overcome, it was essential in honoring the team’s commitment to data sovereignty and self-determination.

Overall, the development team indicated that strong communication, workload distribution and delegation, and continuous evaluation were key internal factors that positively contributed to the platform development:

The communication was really strong between the team despite working from home and not working physically in the same place.

DT2

Participants also noted that having an agile development mindset as a team was helpful, particularly when collaborating with a citizen scientist advisory council consisting of project stakeholders. Digital literacy was also identified as an important component of app usability by the development team. Therefore, it was important for the development team to increase the accessibility of the app by modifying traditional surveys (ie, sociodemographic questions) to include more visuals and graphics while minimizing text where possible (Figure 1).

#### Theme 4: Project Successes and Areas of Improvement

Participants noted three primary moments of success: (1) the development of the household risk management feature, (2) the launch of the progressive web application, and (3) the clearance of the final software tests. One participant noted, in particular, the following:

The constant variation of policies and virus strains resulted in the creation of household risk management, rather than individual risk management, which I think is going to change the way we develop risk management tools for infectious diseases in the future.

DT5

When asked which factors stood out in making the development process successful, participants underscored the importance of the team dynamic. In particular, participants noted the strong leadership, cohesion, ability to problem solve, and their commitment and dedication.

Participants indicated that improving the efficiency of the team overall could be beneficial moving forward, “work on developing a more efficient workload distribution model” (DT4), particularly through simplifying platform features or implementing agile methodology [49] (ie, managing a project by breaking it up into several phases).

One participant noted that the team changed their internal sprint processes and incorporated internal evaluations of ongoing progress:

We had to...rewrite digital application development sprint protocols.

DT5

As described in theme 3, the development team experienced challenges launching health-focused apps, particularly restrictions for COVID-19 apps; thus, the team had to shift to a progressive web application. One participant noted that if they were to restart the project today, they would start with a progressive web application as it would have streamlined project development quite substantially.

#### Theme 5: App Launch Requirements

The participants identified a range of factors that would enable a successful launch. Most of these factors focused on successful uptake; hence, the responses described the importance of awareness and education. When asked to describe some of the factors that would contribute to a successful app launch, participants noted the platform’s adaptability (ie, “rapid adaptation to changing scenarios”), consistent team engagement, and project planning. Strong community leadership and participation was highlighted as an important contributor to the overall success of the platform. One participant noted the following:

The most important factor that I think will contribute to a successful app launch is education and awareness [of the community in which the app is launched].

DT3

Taken together, these results suggested that despite the many challenges faced by the development team, their strong team cohesion, communication, and problem-solving were all essential in the overall success of program development.

### Part B: Pilot Test

A total of 6 stakeholders of the interdisciplinary research team participated in the pilot test, playing the role of citizens or community members and decision makers to simulate real-world conditions. Participant character profiles included a diverse range of ages, occupations, household compositions, COVID-19 risk factors, and food security statuses (Figure 2). Over the course of the 2-week pilot test, there were an average of 7 (SD 3) log-ins per day across an average of 4 users per day (SD 1). The largest proportion (average: 40%, SD 18%) of the daily log-ins occurred in the evening.

In total, 5 (63%) of the 8 participants reported COVID-19 diagnoses, with 21 hospital visits reported over the duration of the pilot test. Among the 8 participants, 2 (25%) were fully vaccinated, 3 (38%) were fully vaccinated with boosters, and 2 (25%) were unvaccinated. Over the duration of the 2-week pilot test, participants reported inconsistently wearing face masks (n=38), making essential shopping trips (n=48), having outdoor visits with other people (n=49), having social gatherings (n=38), and travelling (n=11).

A total of 4 food security requests were placed over the 2-week period, all of which were processed by decision makers using the digital health dashboard. One participant submitted a citizen report, which was processed by a decision maker, and 5 community alerts were issued, all of which were categorized as high urgency.

Results from the postpilot survey are presented in Tables S1 and S2 in Multimedia Appendix 4 for community members and decision makers, respectively.

### Community Members

#### Usability

All (6/6, 100%) participants felt that their avatars accurately reflected their COVID-19 risk, found the consent process clear, and appreciated the anonymity it provided (Figure 1B). All (6/6, 100%) participants reported that the app was easy to navigate and that they could find each feature when needed. Participants found it straightforward to set up their avatars, taking ≤5 minutes. In total, 33% (2/6) of the participants created avatars for multiple household members, while the other 67% (4/6) created avatars for themselves only. According to participants, the ideal frequency of notifications from the app was once per day. No (0/6, 0%) participants reported any issues in using the app.

#### Feature-Specific Feedback

Most (4/6, 67%) of the participants reported that they were comfortable interacting with the platform’s COVID-19 feature, with 33% (2/6) of the participants reporting that they were neither comfortable nor uncomfortable. All (6/6, 100%) participants felt that the recommendations for their COVID-19 risk were clear and easy to understand, though some (2/6, 33%) provided feedback to increase convenience and subsequent usability (eg, readily accessible links or saved log-in information). Nearly all participants (5/6, 83%) were comfortable sharing their vaccination status. Issues such as random logouts and confusion with report creation were raised, though the feature was still found to be easy and clear to use.

Most (5/6, 83%) of the participants used the food security feature, all of which found it “easy” to use. A total of 60% (3/5) of the participants expressed comfort with using this feature, with the 40% (2/5) remaining participants reporting that they were “neither comfortable nor uncomfortable.” Moreover, 60% (3/5) of the participants reported that the response of the decision makers was “neither appropriate nor inappropriate,” while the remaining 40% (2/5) of the participants reported “appropriate” responses. Similarly, half (3/5, 60%) of the participants were “comfortable” revealing their identity when support was needed, while the remainder (2/5, 40%) felt “neither comfortable nor uncomfortable.” When asked about room for improvement, 40% (2/5) of the participants suggested the inclusion of more detailed options for food security requests, 40% (2/5) of the participants reported issues viewing responses to their submitted food requests, and 20% (1/5) of the participants desired additional instructions for interacting with the feature.

Of the 6 participants, 3 (50%) used the citizen reporter feature and felt comfortable doing so. A total of 2 (67%) of the 3 participants found it easy to use, and 1 (33%) participant experienced difficulty during the report submission process, expressing uncertainty regarding the completeness of their submission due to the lack of response. Moreover, 1 (33%) participant reported an appropriate response from the decision maker, while the remaining 2 (67%) participants reported neither appropriate nor inappropriate responses. In total, 2 (67%) of the 3 participants felt comfortable revealing their identity when seeking support, while the third (33%) participant felt neither comfortable nor uncomfortable.

#### Overall Feedback

Participants generally found the app useful, providing clear information and serving as a good resource. The straightforwardness, accessibility, and organization of the app were highlighted along with the easy navigation and relevant tips. However, there were mentions of difficulty in finding where to input information initially and occasional log-in issues requiring password resets. Some (2/6, 33%) participants also mentioned challenges when using the app on a phone, particularly related to limited screen visibility.

### Decision Makers

#### Usability

Decision makers reported on the usability of the digital dashboard, including ease of finding information, navigation, and overall use. Both decision makers found the information used to create their avatars accurate in reflecting their COVID-19 risk. They were satisfied with the clarity of the consent process and believed the app was clear and easy to navigate, ensuring anonymity and accessibility to all features. No issues or improvement suggestions were reported regarding navigability, and participants felt that they knew who to contact with questions regarding the app, their data, or their rights. Both decision makers had no difficulty interacting with notifications, finding them organized and user-friendly.

#### Feature-Specific Feedback

**Figure 3:**
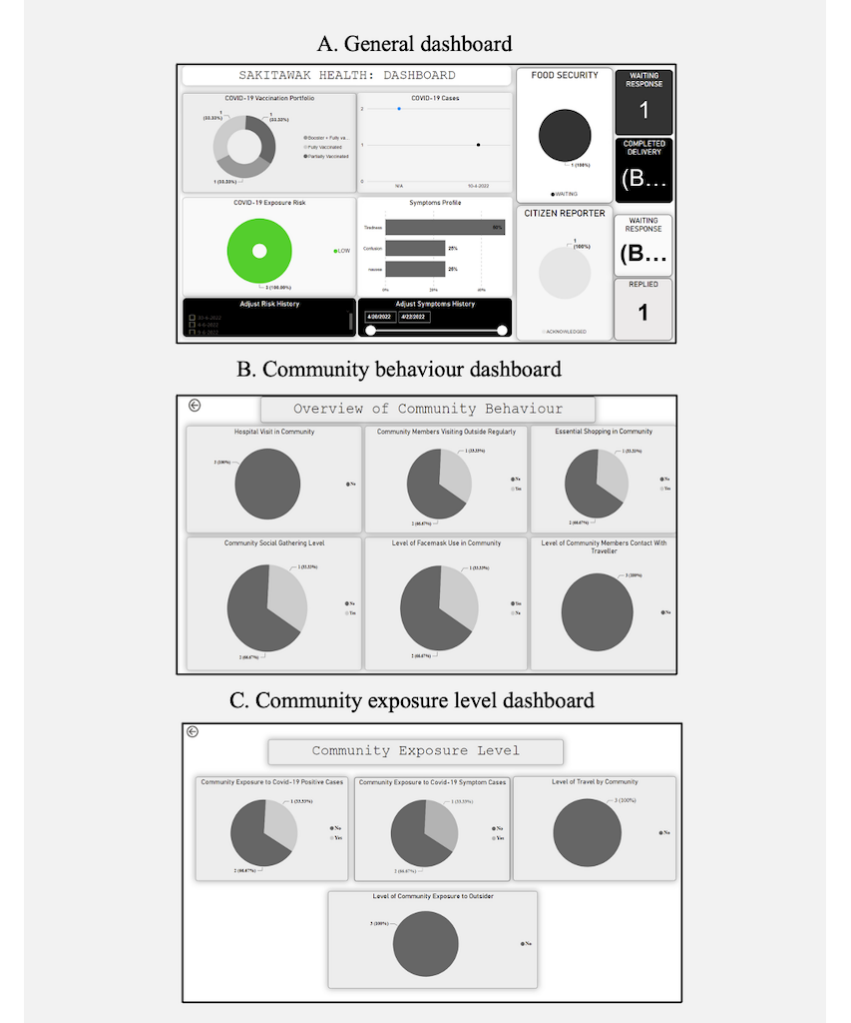
Screenshots of the CO-Away platform interface for decision makers, including the general dashboard, the community behavior dashboard, and the community exposure level dashboard.

Figure 3 shows examples of how community member data were aggregated and visualized for decision makers to review. Participants had a positive experience with the dashboard data visualizations of the dashboard, finding them easy to understand and appreciating the level of control they had over them. Decision makers felt confident in using the presented data to make informed decisions regarding the community’s response to the COVID-19 pandemic. Response rates to individual incidents were relatively short, typically taking less than 5 minutes to complete. No suggestions for improvement or increasing participants’ confidence were provided.

Similar to the COVID-19 feature, both decision makers found the data visualizations easy to understand and appreciated the appearance and level of control over them. Decision makers expressed confidence in using the presented data for informed decision-making regarding the COVID-19 pandemic response. Response rates for individual incidents were deemed prompt, with completion times less than 5 minutes. No suggestions for improving confidence or the visualizations were given.

Only 1 of the 2 decision makers interacted with the citizen reporter feature, reported that the data visualizations were easy to understand, and appreciated their appearance and level of control. The decision maker expressed confidence in using the presented data for informed decision-making, and the participant considered the response times for individual incidents, completed in less than 5 minutes, to be appropriate. No suggestions for improving the visualizations or participant confidence were given.

#### Overall Feedback

Participants had a positive overall experience with the platform, with a participant suggesting the addition of reference material, such as a brief presentation or a short video, for future use. The food security section was found to be easy to navigate and straightforward, providing users with simplified access. However, there were uncertainties regarding the ability to delete requests and whether citizens could contact local decision makers and/or food services for questions or concerns, as a participant did not receive a notification when attempting to do so.

## Discussion

### Principal Findings

Evaluation of the CO-Away digital health platform enabled systematic capture of the development team’s perspective on the overall development process as well as the testing of platform usability from multiple stakeholders simulating real-world scenarios before finalizing platform features. This evaluation demonstrated that while the final digital health platform met its original objectives, it had to be expanded to accommodate the evolving needs of potential users and the COVID-19 pandemic regulations. Strong team cohesion, communication, and problem-solving were essential to the overall success of platform development. The pilot test found that participants reported positive experiences interacting with the platform and found its features relatively easy to use, with a few community members reporting challenges interacting with the interface. Users in the decision maker role found the data visualizations intuitive in helping them to understand the information.

### Relevance and Application of Findings

The developmental evaluation findings will not only influence the decisions of both research and development teams going forward, but the process described for conducting an assessment of the CO-Away development phase also importantly advances a replicable methodology that can inform empirical development, implementation, and evaluation of digital health platforms [6]. The field of digital health evaluation is rapidly evolving; therefore, emerging research on innovations in digital technology and evaluation practices to assess impact and utility [28,38,50-52] have emerged over the past 5 years. The World Health Organization [28] has released guidelines for monitoring and evaluating digital health interventions, with many existing mixed methods (ie, qualitative focus groups and quantitative surveys) being applied to digital health platform evaluations. However, there is little guidance on how and when to apply these evaluation methods in the scheme of a digital health project [53,54]. In particular, there is a lack of peer-reviewed literature describing the evaluation of the development of evidence-based digital health platforms.

When working with marginalized populations, such as Indigenous or global south communities, evaluation of the development process is a critical step in ensuring that a digital platform is safe, accessible, and not widening existing inequities. Specific considerations related to data ownership, cultural competency, and privacy must be built into the digital platform development protocol and rigorously tested [34,52,53]. Thus, this developmental evaluation was designed by initially referring to existing evaluation literature to inform the key evaluation questions and study approach [38,44-47]. Given the dearth of evidence on *how* to practically adapt and apply digital evaluation methods [53], our study aimed to address this gap by implementing an innovative mixed methods approach informed by previous evaluation work in Indigenous communities [34] and evaluation literature [29,44-47,55-59].

The use of role-play has been demonstrated in other contexts [55-59] where the application of a digital intervention or program can be simulated before public deployment. For example, during the evaluation of a digital platform designed to improve the reproductive health of adolescents in Rwanda, a role-play exercise was used during the prototyping stage of development to evaluate the end-to-end user experience [29]. During the role-play exercise, participants were provided with mock scenarios and roles to help the design team understand how users interacted with the interface during the improvised scenario-specific performances. Similar to the approach used in this developmental evaluation, feedback was requested from participants following the role-play exercise to gain an understanding of user experience and areas of improvement. This step is essential not only when working with marginalized communities but also for launching digital platforms focused on sensitive health issues, that is, when potential risks are heightened, thereby requiring more rigorous testing protocols. Identifying qualified participants for role-play can be a challenge in research studies; therefore, this evaluation uniquely used an interdisciplinary team to conduct the exercise during the development phase to minimize commonly reported challenges with role-play and simulate a real-world scenario before the public launch [56].

It was through this evaluation that our internal testing of the platform generated key learnings that informed specific project decisions, including integrating the research team more cohesively with the development team. In academic research, a team of external technology vendors is commonly used to develop digital platforms. As a result, the external development team is often not embedded in the research and evaluation process. In order to effectively evaluate the digital development process, our team identified the need to involve a data scientist as part of both the development and research teams going forward (whereas previously, only a researcher was liaising with the development team). Including a common computer programmer or developer in both teams improved communication and coordination during the development process. By bridging these traditionally distinct teams, we have been able to maximize cross-disciplinary collaboration, with research and practice informing development decisions. This integration also proved instrumental in aligning the platform’s features with users’ needs and developmental feasibility, that is, integrating interdisciplinarity into the development process.

In recognizing the potential of concurrent health domain expertise and software development skills, we initiated a health system training for all computer programmers [60,61]. This training contributed to increasing interdisciplinary understanding and communication and was deemed necessary for the creation of user-centred CO-Away feature development, which considered distinct risks that could be introduced with AI systems [36,37]. This training not only supports the programmers’ comprehension of the health context but also provides them with the ability to envision the platform’s functionalities from the user’s perspective. This is a key step in both co-designing digital infrastructure as well as designing human-centered AI [35,62], which is a gap in the current literature [63-66]. In general, this approach resulted in digital health platform features that were user-friendly, that is, an improved user experience, which is critical to the success of platform use and implementation [64,67].

The developmental evaluation also changed development sprint processes [49,68], which paved the way for shorter sprints with a quick internal evaluation of ongoing progress. Software development sprints, which are part of the agile coding process, are implemented as part of the agile methodology, an iterative project management framework that breaks projects down into several dynamic phases, commonly known as “sprints” [49]. When the team started the CO-Away development process, sprints were approximately 4 to 5 months, but the developmental evaluation findings informed our decision to shorten the sprints to 3 to 4 months to enable faster internal testing and modification of the digital platform. This decision has allowed the software development team to pivot quickly when necessary, responding to stakeholder feedback and incorporating changes to the digital platform iteratively.

The evaluation provided noteworthy insights into the dynamics of our team, particularly the role that strong communication played throughout the development process. The COVID-19 pandemic presented a unique set of challenges that required adaptive strategies [69-71]. Despite the physical distancing mandates, our team managed to function cohesively, leveraging digital tools, regular virtual meetings, and transparent channels of communication, that is, open discussion of weekly updates and key decisions accessible to all team members on a virtual portal. The development process not only involved remote hiring of our team [72] but also remote functioning [73] and retention of our team even after the pandemic restrictions ended, lessons which we implemented into the continuous functioning of our development processes. For instance, we currently work in a hybrid setting [74], which leverages the flexibility of virtual work as well as in-person brainstorming, a system that has further improved communication and collaboration with research and development teams.

Perhaps the defining element that shaped the development process of the CO-Away platform was the complexities that arose with its COVID-19–specific focus. The evolving nature of the coronavirus [75-77], coupled with the rapidly changing landscape of pandemic management policies [78-80], presented a constant need for flexibility and adaptability. The virus’s mutations necessitated continuous updates to the platform’s algorithms to ensure that users would have access to the most up-to-date information. Similarly, the ever-changing policies surrounding lockdowns [81,82], vaccine requirements [83,84], and mask mandates [85,86] required the platform’s features to be responsive to shifting guidelines [6]. Navigating these complexities highlighted the importance of further adapting the agile methodology [49,68] throughout the platform design and development process to accommodate the changing COVID-19 landscape.

The role-play pilot test highlighted the effectiveness of adhering to user-centered design principles throughout the development of the CO-Away digital health platform. The stakeholders who participated in the pilot reported overall positive reactions to the platform avatars, transparent consent procedures, and intuitive navigation—aspects that reiterated the importance of tailoring the platform design to users’ needs and ensuring a user-friendly experience [64,67]. While participants generally found the app user-friendly, their feedback on minor obstacles, such as entering initial information and sporadic log-in difficulties, highlighted the importance of iterative improvement during the development process. This constructive feedback provided an opportunity for the development team to adapt CO-Away to meet the needs of users.

The mixed feedback regarding participants’ comfort levels when interacting with the platform features reveals the importance of balancing interactivity with user comfort. For instance, while some participants were comfortable engaging with various features and sharing personal information for support, others expressed hesitancy. This finding highlights the significance of providing users with control over their data, a critical aspect of data sovereignty [32] that has been built into platform development after this vital input.

While we did not observe marked differences in the way participants engaged with the platform based on the assigned participant roles, it is worth noting that there were differences in platform use between decision makers and community members. These variations align with expectations stemming from the differing ways these distinct user groups engage with the platform’s features. For instance, decision makers are less focused on their own privacy as they are not sharing data, while the community members are sharing data. We anticipate that various community members will interact with and use the app according to their specific circumstances and needs. As such, the potential differences in app use based on community member roles will be further explored in the future during the external community pilot phase.

Finally, the digital health dashboard feature of CO-Away offered decision makers with valuable tools for informed decision-making, especially within the context of COVID-19 response and community management. The pilot decision makers appreciated the usability of the platform and expressed confidence in using the data visualizations for informed decision-making. However, it is important to recognize that although some real-world scenarios were simulated in the pilot, a larger external community pilot will require more complex decision-making due to the increased scale of implementation. However, the key goal of the pilot was to test the functionality of a cloud-based digital health dashboard to engage with community members and send real-time alerts, features that were successfully tested and functionality that was confirmed in the evaluation of the pilot. Real-world policy changes using real-time big data would require stakeholder buy-in and addressing nuanced matters, such as food security and citizen reporter reports, demanding sensitivity and community-wide consideration. Further exploration of these dynamics will be carried out during our upcoming external community pilot.

### Key Guidelines for Digital Health Platform Development

On the basis of the findings of this developmental evaluation of a digital health platform development, we suggest the following guidelines:

1. Integration of research and development teams is key to the success of digital health platforms, where the software developers are provided an opportunity to understand scientific goals, and the research stakeholders are made privy to cloud computing challenges.
2. Conducting a developmental evaluation after the completion of a prototype facilitates the incorporation of research and development team perspectives empirically into the iterative digital development process before a product is tested widely in the community.
3. Conducting an internal role-play pilot simulating real-world conditions is critical to test not only the functionality of digital health platforms but also the nuanced perceptions of potential users, which will be essential for successful community implementation.

### Strengths and Limitations

This developmental evaluation has several notable strengths. First, we deployed a mixed methods approach to data collection and analysis; open-ended survey questions to capture participant perceptions of the platform and its usability and CO-Away use metrics data provided an objective assessment of platform engagement. The integration of both quantitative and qualitative data collection methods enriches the findings generated by the evaluation. Second, the inclusion of development team surveys allowed for an in-depth understanding not only of CO-Away’s developmental process but also offered valuable insights that have and will continue to inform improvements in the development processes. Finally, the use of “character roles” was used to simulate a community pilot test. Although not a substitute for a real-world pilot, this approach served as a valuable first step for exploring real-world simulations using varied user interactions and experiences, an approach that will shape a future external community pilot. Providing fictional role-play characters through this remote evaluation also avoided potential issues, such as staying in character [56]. There were also several study limitations. The participation of the stakeholders in the pilot test may have affected the nature of the issues reported, that is, more focus on app function than readability. In order to minimize potential bias, the evaluation questions were blinded, and the digital platform development team did not participate in the pilot test. Moreover, the stakeholders who participated in the pilot did not analyze the evaluation data. The use of “character roles,” while insightful, does not capture all user scenarios that would arise in a real-world setting. As such, it is possible that certain user interactions and challenges were not captured in this evaluation and will be explored during our external community pilot. Notably, the sample size for each population was small, so caution should be taken when interpreting or comparing percentages. Another limitation pertains to the digital literacy of participants. While the pilot participants did not have previous experience using the platform, it is possible that few issues were reported due to the inherent high digital literacy of the interdisciplinary stakeholders. In future digital evaluations, it would be imperative to not only capture the digital literacy of platform users but also to enable the increase of digital literacy through innovative digital literacy programs [87].

### Conclusions

The evaluation of digital health platform development is critical for the success of not only platform functionality but also eventual implementation and scale-up. The innovative mixed methods approach applied in this evaluation combines both perspectives of the development team as well as a real-world simulation of platform users to extend key guidelines for digital health platform development. For sensitive community health issues, role-play simulation and testing before public launch can avoid potential risks and improve user experience. The evaluation informed several key decisions of the digital health platform development, including integrating the research team more cohesively with the development team, which resulted in a data scientist being part of both teams going forward. Another key development process decision was to integrate more interdisciplinarity into the development process by providing health system training to computer programmers and shortening development sprints. Combined with more efficient sprints, the role-play exercise importantly enabled improvements to the log-in process and the user interface ahead of public deployment.

## Appendix

Multimedia Appendix 1 Development survey.

Multimedia Appendix 2 Postpilot survey—community member.

Multimedia Appendix 3 Postpilot survey—decision maker.

Multimedia Appendix 4 Evaluation survey results.

## Abbreviations

AI: artificial intelligence

## References

[1] Alokaily F (2021). COVID-19 global health crisis. Saudi Med J.

[2] (2024). Noncommunicable diseases. World Health Organization.

[3] Lawrence M, Homer-Dixon T, Janzwood S, Rockstrom J, Renn O, Donges JF (2023). Global polycrisis: the causal mechanisms of crisis entanglement. SSRN J.

[4] Katapally TR (2020). A global digital citizen science policy to tackle pandemics like COVID-19. J Med Internet Res.

[5] Sheikh A, Anderson M, Albala S, Casadei B, Franklin BD, Richards M, Taylor D, Tibble H, Mossialos E (2021). Health information technology and digital innovation for national learning health and care systems. Lancet Digit Health.

[6] Katapally TR, Ibrahim ST (2023). Digital health dashboards for decision-making to enable rapid responses during public health crises: replicable and scalable methodology. JMIR Res Protoc.

[7] Katapally TR (2019). The SMART framework: integration of citizen science, community-based participatory research, and systems science for population health science in the digital age. JMIR Mhealth Uhealth.

[8] Stoumpos AI, Kitsios F, Talias MA (2023). Digital transformation in healthcare: technology acceptance and its applications. Int J Environ Res Public Health.

[9] Budd J, Miller BS, Manning EM, Lampos V, Zhuang M, Edelstein M, Rees G, Emery VC, Stevens MM, Keegan N, Short MJ, Pillay D, Manley E, Cox IJ, Heymann D, Johnson AM, McKendry RA (2020). Digital technologies in the public-health response to COVID-19. Nat Med.

[10] Rosenlund M, Kinnunen UM, Saranto K (2023). The use of digital health services among patients and citizens living at home: scoping review. J Med Internet Res.

[11] Jnr BA (2020). Use of telemedicine and virtual care for remote treatment in response to COVID-19 pandemic. J Med Syst.

[12] Batko K, Ślęzak A (2022). The use of big data analytics in healthcare. J Big Data.

[13] Dash S, Shakyawar SK, Sharma M, Kaushik S (2019). Big data in healthcare: management, analysis and future prospects. J Big Data.

[14] Burton L, Rush KL, Smith MA, Davis S, Rodriguez Echeverria P, Suazo Hidalgo L, Görges M (2022). Empowering patients through virtual care delivery: qualitative study with micropractice clinic patients and health care providers. JMIR Form Res.

[15] Kuwabara A, Su S, Krauss J (2020). Utilizing digital health technologies for patient education in lifestyle medicine. Am J Lifestyle Med.

[16] Mair JL, Salamanca-Sanabria A, Augsburger M, Frese BF, Abend S, Jakob R, Kowatsch T, Haug S (2023). Effective behavior change techniques in digital health interventions for the prevention or management of noncommunicable diseases: an umbrella review. Ann Behav Med.

[17] Riso B, Tupasela A, Vears DF, Felzmann H, Cockbain J, Loi M, Kongsholm NC, Zullo S, Rakic V (2017). Ethical sharing of health data in online platforms - which values should be considered?. Life Sci Soc Policy.

[18] Mosa AS, Yoo I, Sheets L (2012). A systematic review of healthcare applications for smartphones. BMC Med Inform Decis Mak.

[19] Langford AT, Solid CA, Scott E, Lad M, Maayan E, Williams SK, Seixas AA (2019). Mobile phone ownership, health apps, and tablet use in US adults with a self-reported history of hypertension: cross-sectional study. JMIR Mhealth Uhealth.

[20] Kaplan WA (2006). Can the ubiquitous power of mobile phones be used to improve health outcomes in developing countries?. Global Health.

[21] Willis VC, Thomas Craig KJ, Jabbarpour Y, Scheufele EL, Arriaga YE, Ajinkya M, Rhee KB, Bazemore A (2022). Digital health interventions to enhance prevention in primary care: scoping review. JMIR Med Inform.

[22] Haleem A, Javaid M, Singh RP, Suman R (2021). Telemedicine for healthcare: capabilities, features, barriers, and applications. Sens Int.

[23] Shahid N, Rappon T, Berta W (2019). Applications of artificial neural networks in health care organizational decision-making: a scoping review. PLoS One.

[24] Vazquez-Ingelmo A, Garcia-Penalvo FJ, Theron R (2019). Information dashboards and tailoring capabilities - a systematic literature review. IEEE Access.

[25] Kostkova P (2018). Disease surveillance data sharing for public health: the next ethical frontiers. Life Sci Soc Policy.

[26] Bouabida K, Lebouché B, Pomey MP (2022). Telehealth and COVID-19 pandemic: an overview of the telehealth use, advantages, challenges, and opportunities during COVID-19 pandemic. Healthcare (Basel).

[27] Abernethy A, Adams L, Barrett M, Bechtel C, Brennan P, Butte A, Faulkner J, Fontaine E, Friedhoff S, Halamka J, Howell M, Johnson K, Long P, McGraw D, Miller R, Lee P, Perlin J, Rucker D, Sandy L, Savage L, Stump L, Tang P, Topol E, Tuckson R, Valdes K (2022). The promise of digital health: then, now, and the future. NAM Perspect.

[28] (2016). Monitoring and evaluating digital health interventions: a practical guide to conducting research and assessment. World Health Organization.

[29] Ippoliti N, Sekamana M, Baringer L, Hope R (2021). Using human-centered design to develop, launch, and evaluate a national digital health platform to improve reproductive health for Rwandan youth. Glob Health Sci Pract.

[30] Beynon F, Guérin F, Lampariello R, Schmitz T, Tan R, Ratanaprayul N, Tamrat T, Pellé KG, Catho G, Keitel K, Masanja I, Rambaud-Althaus C (2023). Digitalizing clinical guidelines: experiences in the development of clinical decision support algorithms for management of childhood illness in resource-constrained settings. Glob Health Sci Pract.

[31] Tamrat T, Chandir S, Alland K, Pedrana A, Taighoon Shah M, Footitt C, Snyder J, Ratanaprayul N, Arif Siddiqi D, Nazneen N, Fitria Syah I, Wong R, Lubell-Doughtie P, Dwi Utami A, Anwar K, Ali H, Labrique AB, Say L, Shankar AH, Livingston Mehl G (2022). Digitalization of routine health information systems: Bangladesh, Indonesia, Pakistan. Bull World Health Org.

[32] Bhawra J (2022). Decolonizing digital citizen science: applying the bridge framework for climate change preparedness and adaptation. Societies.

[33] Hummel P, Braun M, Tretter M, Dabrock P (2021). Data sovereignty: a review. Big Data Soc.

[34] Bhawra J, Buchan MC, Green B, Skinner K, Katapally TR (2022). A guiding framework for needs assessment evaluations to embed digital platforms in partnership with Indigenous communities. PLoS One.

[35] Bingley WJ, Curtis C, Lockey S, Bialkowski A, Gillespie N, Haslam SA, Ko RK, Steffens N, Wiles J, Worthy P (2023). Where is the human in human-centered AI? Insights from developer priorities and user experiences. Comput Hum Behav.

[36] Ozmen Garibay O, Winslow B, Andolina S, Antona M, Bodenschatz A, Coursaris C, Falco G, Fiore SM, Garibay I, Grieman K, Havens JC, Jirotka M, Kacorri H, Karwowski W, Kider J, Konstan J, Koon S, Lopez-Gonzalez M, Maifeld-Carucci I, McGregor S, Salvendy G, Shneiderman B, Stephanidis C, Strobel C, Ten Holter C, Xu W (2023). Six human-centered artificial intelligence grand challenges. Int J Hum Comput Interact.

[37] Chen Y, Clayton EW, Novak LL, Anders S, Malin B (2023). Human-centered design to address biases in artificial intelligence. J Med Internet Res.

[38] Murray E, Hekler EB, Andersson G, Collins LM, Doherty A, Hollis C, Rivera DE, West R, Wyatt JC (2016). Evaluating digital health interventions: key questions and approaches. Am J Prev Med.

[39] Sanz MF, Acha BV, García MF (2021). Co-design for people-centred care digital solutions: a literature review. Int J Integr Care.

[40] (2021). The impact of COVID-19 on rural and remote mental health and substance use. Mental Health Commission of Canada.

[41] Huyser KR, Yellow Horse AJ, Collins KA, Fischer J, Jessome MG, Ronayne ET, Lin JC, Derkson J, Johnson-Jennings M (2022). Understanding the associations among social vulnerabilities, indigenous peoples, and COVID-19 cases within Canadian health regions. Int J Environ Res Public Health.

[42] Rêgo F, Portela F, Santos MF (2019). Towards PWA in healthcare. Procedia Comput Sci.

[43] Bhawra J, Elsahli N, Patel J (2024). Applying digital technology to understand human experiences of climate change impacts on food security and mental health: scoping review. JMIR Public Health Surveill.

[44] Dozois E, Langlois M, Blanchet-Cohen N (2010). DE 201: a practitioner’s guide to developmental evaluation. The J.W. McConnell Family Foundation and the International Institute for Child Rights and Development.

[45] Patton MQ (2021). Emergent developmental evaluation developments. J Multidiscip Eval.

[46] Patton MQ (2008). Utilization-Focused Evaluation.

[47] Patton MQ (2010). Developmental Evaluation: Applying Complexity Concepts to Enhance Innovation and Use.

[48] (2018). Tri-council policy statement: ethical conduct for research involving humans. Canadian Institutes of Health Research, Natural Sciences and Engineering Research Council of Canada, and Social Sciences and Humanities Research Council.

[49] Nazir S, Price B, Surendra NC, Kopp K (2022). Adapting agile development practices for hyper-agile environments: lessons learned from a COVID-19 emergency response research project. Inf Technol Manag.

[50] Kolasa K, Kozinski G (2020). How to value digital health interventions? A systematic literature review. Int J Environ Res Public Health.

[51] Bashi N, Fatehi F, Mosadeghi-Nik M, Askari MS, Karunanithi M (2020). Digital health interventions for chronic diseases: a scoping review of evaluation frameworks. BMJ Health Care Inform.

[52] Labrique AB, Wadhwani C, Williams KA, Lamptey P, Hesp C, Luk R, Aerts A (2018). Best practices in scaling digital health in low and middle income countries. Global Health.

[53] Guo C, Ashrafian H, Ghafur S, Fontana G, Gardner C, Prime M (2020). Challenges for the evaluation of digital health solutions-a call for innovative evidence generation approaches. NPJ Digit Med.

[54] Wang T, Giunti G, Goossens R, Melles M (2024). Timing, indicators, and approaches to digital patient experience evaluation: umbrella systematic review. J Med Internet Res.

[55] Skoura-Kirk E, Brown S, Mikelyte R (2020). Playing its part: an evaluation of professional skill development through service user-led role-plays for social work students. Soc Work Educ.

[56] Seland G, Kurosu M (2009). Empowering end users in design of mobile technology using role play as a method: reflections on the role-play conduction. Human Centered Design.

[57] Elhilu AH, El-Setouhy M, Mobarki AS, Abualgasem MM, Ahmed MA (2023). Peer role-play simulation: a valuable alternative to bedside teaching during the COVID-19 pandemic. Adv Med Educ Pract.

[58] Xu L, Liu W, Jiang X, Li Y (2023). Impact of using peer role-playing on the clinical skills performance of pediatric trainees. BMC Med Educ.

[59] Reiff M, Spiegel A, Williams E, Ramesh B, Madabhushi S, Bvunzawabaya B (2022). From role-play to real life: using gatekeeper skills in real-world situations. J Coll Stud Ment Health.

[60] Moreb M, Mohammed TA, Bayat O (2020). A novel software engineering approach toward using machine learning for improving the efficiency of health systems. IEEE Access.

[61] Ibeneme S, Karamagi H, Muneene D, Goswami K, Chisaka N, Okeibunor J (2022). Strengthening health systems using innovative digital health technologies in Africa. Front Digit Health.

[62] Mhlanga D (2022). Human-centered artificial intelligence: the superlative approach to achieve sustainable development goals in the fourth industrial revolution. Sustainability.

[63] Grindell C, Coates E, Croot L, O'Cathain A (2022). The use of co-production, co-design and co-creation to mobilise knowledge in the management of health conditions: a systematic review. BMC Health Serv Res.

[64] Nusir M, Rekik M (2022). Systematic review of co-design in digital health for COVID-19 research. Univers Access Inf Soc.

[65] Slattery P, Saeri AK, Bragge P (2020). Research co-design in health: a rapid overview of reviews. Health Res Policy Syst.

[66] Tremblay M, Hamel C, Viau-Guay A, Giroux D (2022). User experience of the co-design research approach in eHealth: activity analysis with the course-of-action framework. JMIR Hum Factors.

[67] Madanian S, Nakarada-Kordic I, Reay S, Chetty T (2023). Patients' perspectives on digital health tools. PEC Innov.

[68] Kortum F, Klünder J, Brunotte W, Schneider K (2019). Sprint performance forecasts in agile software development - the effect of futurespectives on team-driven dynamics. Proceedings of the 31st International Conference on Software Engineering and Knowledge Engineering.

[69] Graziani AR, Botindari L, Menegatti M, Moscatelli S (2023). Adaptive coping strategies at the time of COVID-19: the role of social and general trust. Int J Environ Res Public Health.

[70] Maison D, Jaworska D, Adamczyk D, Affeltowicz D (2021). The challenges arising from the COVID-19 pandemic and the way people deal with them. A qualitative longitudinal study. PLoS One.

[71] Fegert JM, Vitiello B, Plener PL, Clemens V (2020). Challenges and burden of the Coronavirus 2019 (COVID-19) pandemic for child and adolescent mental health: a narrative review to highlight clinical and research needs in the acute phase and the long return to normality. Child Adolesc Psychiatry Ment Health.

[72] Kowalski G, Ślebarska K (2022). Remote working and work effectiveness: a leader perspective. Int J Environ Res Public Health.

[73] Henke JB, Jones SK, O'Neill TA (2022). Skills and abilities to thrive in remote work: what have we learned. Front Psychol.

[74] Krajčík M, Schmidt DA, Baráth M (2023). Hybrid work model: an approach to work–life flexibility in a changing environment. Adm Sci.

[75] Sharma A, Ahmad Farouk I, Lal SK (2021). COVID-19: a review on the novel coronavirus disease evolution, transmission, detection, control and prevention. Viruses.

[76] Sun J, He WT, Wang L, Lai A, Ji X, Zhai X, Li G, Suchard MA, Tian J, Zhou J, Veit M, Su S (2020). COVID-19: epidemiology, evolution, and cross-disciplinary perspectives. Trends Mol Med.

[77] - (2021). Effectiveness of public health measures in reducing the incidence of covid-19, SARS-CoV-2 transmission, and covid-19 mortality: systematic review and meta-analysis. BMJ.

[78] Cavalcante de Oliveira AP, Galante ML, Maia LS, Craveiro I, da Silva AP, Fronteira I, Chança R, Cometto G, Ferrinho P, Dal Poz M (2023). Implementation of policy and management interventions to improve health and care workforce capacity to address the COVID-19 pandemic response: a systematic review. Hum Resour Health.

[79] (2022). First lessons from government evaluations of COVID-19 responses: a synthesis. Organisation for Economic Co-operation and Development.

[80] Sigahi TF, Kawasaki BC, Bolis I, Morioka SN (2021). A systematic review on the impacts of Covid-19 on work: contributions and a path forward from the perspectives of ergonomics and psychodynamics of work. Hum Factors Ergon Manuf.

[81] Yanovskiy M, Socol Y (2022). Are lockdowns effective in managing pandemics?. Int J Environ Res Public Health.

[82] Panchal U, Salazar de Pablo G, Franco M, Moreno C, Parellada M, Arango C, Fusar-Poli P (2023). The impact of COVID-19 lockdown on child and adolescent mental health: systematic review. Eur Child Adolesc Psychiatry.

[83] Bardosh K, de Figueiredo A, Gur-Arie R, Jamrozik E, Doidge J, Lemmens T, Keshavjee S, Graham JE, Baral S (2022). The unintended consequences of COVID-19 vaccine policy: why mandates, passports and restrictions may cause more harm than good. BMJ Glob Health.

[84] Maquiling A, Jeevakanthan A, Ho Mi Fane B (2023). The effect of vaccine mandate announcements on vaccine uptake in Canada: an interrupted time series analysis. Vaccine.

[85] Zhang YS, Young Leslie H, Sharafaddin-Zadeh Y, Noels K, Lou NM (2021). Public health messages about face masks early in the COVID-19 pandemic: perceptions of and impacts on Canadians. J Community Health.

[86] He L, He C, Reynolds TL, Bai Q, Huang Y, Li C, Zheng K, Chen Y (2021). Why do people oppose mask wearing? A comprehensive analysis of U.S. tweets during the COVID-19 pandemic. J Am Med Inform Assoc.

[87] Buchan MC, Bhawra J, Katapally TR (2023). Navigating the digital world: development of an evidence-based digital literacy program and assessment tool for youth. Smart Learn Environ.

