# Supplementary material for: Application of an Innovative Methodology to Build Infrastructure for Digital Transformation of Health Systems: Developmental Program Evaluation
Source: JMIR Form Res. 2025 Apr 17;9:e53339. doi: 10.2196/53339 (PMC12046263; doi:10.2196/53339)
Supplement: Multimedia Appendix 1 [file formative_v9i1e53339_app1.docx]

**Appendix A: Development survey**

Q1 Can you briefly explain your role on the project?

Q2 To what extent does the final app prototype look like what was originally planned?

Q3 How did changing government policies (e.g., mask mandate, vaccine passport, travel restrictions, business closures, social distancing, etc.) impact the app development or specific app features ?

Q4 How has the intended target audience for the app changed?

Q5 When you think back to the development of this project, what challenges did you and your team face?

Q6 What external factors influenced the app development? (e.g., COVID-19, experience with partnerships, funding)

Q7 What internal factors influenced the app development? (e.g., organizational structure, communication, etc.)

Q9 Can you describe some of factors that would contribute to a successful app launch?

Q10 a) Can you think of a time where you and your team had a big success? Or when things were running quite smoothly for this project? Please describe.
b) Were there any factors that stood out to making this time successful?

Q11 If you were to restart app development today, how might you approach things differently?

Q12 What additional resources might have been beneficial (e.g., funding, time, partnerships, staff)?

Q13 What impact do you think this app (as it is now) will have on: Food insecurity, COVID-19 management, and citizen reporting?

Q14 Has the potential for impact on citizens changed with the latest app revisions? How so?
