# Supplementary material for: Application of an Innovative Methodology to Build Infrastructure for Digital Transformation of Health Systems: Developmental Program Evaluation
Source: JMIR Form Res. 2025 Apr 17;9:e53339. doi: 10.2196/53339 (PMC12046263; doi:10.2196/53339)
Supplement: Multimedia Appendix 2 [file formative_v9i1e53339_app2.docx]

**Appendix B: Post-pilot survey – Community member**

1. How well do you feel that the information used to create your avatar accurately reflects your risk of contracting COVID-19?

- Not accurately at all
- Somewhat accurately
- Accurately

1. After registering for the app, I feel that...

|  | Agree (1) | Somewhat agree (3) | Somewhat disagree (4) | Disagree (2) |
| --- | --- | --- | --- | --- |
| The consent process was clear |  |  |  |  |
| The app was easy to navigate |  |  |  |  |
| I could find each app feature when needed |  |  |  |  |
| My identity is anonymous |  |  |  |  |
| I know who to contact if I have any questions for using the app or regarding my data or rights |  |  |  |  |

*Display This Question:*

*If After registering for the app, I feel that... = Disagree*

1. Please describe any issues you experienced or suggestions for improvement:
2. How many notifications would you prefer to receive from the app?

- 2-3 times per day
- Once per day
- 2-3 times per week
- Once per week
- It depends. Please specify:

1. How long (in minutes) did it take you to complete your household avatars?
2. How many people did you create an avatar for?
3. Did you feel that this process was too long?

- No
- Yes

**The following 5 questions are specific to the COVID risk feature in the app**

1. How easy did you find this feature to use?

- Easy
- Neither easy nor difficult
- Difficult

*Display This Question:*

*If How easy did you find this feature to use? = Difficult*

1. Please describe any issues you experienced or suggestions for improvement:
2. How comfortable were you interacting (e.g., reporting your social encounters) with this feature?

- Comfortable
- Neither comfortable nor uncomfortable
- Uncomfortable

1. Recommendations for my COVID risk were clear and easy to understand

- No
- Yes

*Display This Question:*

*If Recommendations for my COVID risk were clear and easy to understand = No*

1. Please describe any issues you experienced
2. Which elements of this feature could be improved, and how?
3. How comfortable were you sharing your vaccination status?

- Comfortable
- Neither comfortable nor uncomfortable
- Uncomfortable

**The following 6 questions are specific to the food security feature in the app**

1. Did you use the food security feature?

- No
- Yes

*Skip To: End of Block If Did you use the food security feature? = No*

1. How easy did you find this feature to use?

- Easy
- Neither easy nor difficult
- Difficult

*Display This Question:*

*If How easy did you find this feature to use? = Difficult*

1. Please describe any issues you experienced
2. How comfortable were you using this feature (e.g., taking a photo of your fridge)?

- Comfortable
- Neither comfortable nor uncomfortable
- Uncomfortable

1. Which elements of this feature could be improved, and how?
2. How appropriate/sensitive was the response on the decision-makers end?

- Appropriate
- Somewhat appropriate
- Neither appropriate nor inappropriate
- Somewhat inappropriate
- Inappropriate

1. How comfortable were you revealing your identity when support was needed?

- Comfortable
- Neither comfortable nor uncomfortable
- Uncomfortable

**The following 6 questions are specific to the Citizen reporter feature in the app**

1. Did you use the citizen reporter feature?

- No
- Yes

*Skip To: End of Block If Did you use the citizen reporter feature? = No*

1. How easy did you find this feature to use?

- Easy
- Neither easy nor difficult
- Difficult

*Display This Question:*

*If How easy did you find this feature to use? = Difficult*

1. Please describe any issues you experienced
2. How comfortable were you interacting (e.g., reporting your incident, uploading photos) with this feature?

- Comfortable
- Neither comfortable nor uncomfortable
- Uncomfortable

1. Which elements of this feature could be improved, and how?
2. How appropriate/sensitive was the response on the decision-makers end?

- Appropriate
- Somewhat appropriate
- Neither appropriate nor inappropriate
- Somewhat inappropriate
- Inappropriate

1. How comfortable were you revealing your identity when support was needed?

- Comfortable
- Neither comfortable nor uncomfortable
- Difficult

1. Are there ways we could improve this feature?

**General**

1. Is there any other feedback that you would like to share about your experience using the app?
