# Supplementary material for: Application of an Innovative Methodology to Build Infrastructure for Digital Transformation of Health Systems: Developmental Program Evaluation
Source: JMIR Form Res. 2025 Apr 17;9:e53339. doi: 10.2196/53339 (PMC12046263; doi:10.2196/53339)
Supplement: Multimedia Appendix 3 [file formative_v9i1e53339_app3.docx]

**Appendix C: Post-pilot survey – Decision-maker**

1. Do you feel that the information used to create your avatar accurately reflects your personal risk of contracting COVID-19?

- No
- Yes

*Display This Question:*

*If Do you feel that the information used to create your avatar accurately reflects your personal ris... = No*

1. What pieces of information are missing? What else would you like to share about your risk?
2. After registering for the app, I feel that...

|  | Agree (1) | Somewhat agree (3) | Somewhat disagree (4) | Disagree (2) |
| --- | --- | --- | --- | --- |
| The consent process was clear (1) |  |  |  |  |
| The app was easy to navigate (2) |  |  |  |  |
| I could find each app feature when needed (3) |  |  |  |  |
| My identity is anonymous (4) |  |  |  |  |
| I know who to contact if I have any questions for using the app or regarding my data or rights (5) |  |  |  |  |

*Display This Question:*

*If After registering for the app, I feel that... = Somewhat disagree*

*And After registering for the app, I feel that... = Disagree*

1. Please describe any issues you experienced
2. I understand how to interact with a notification

- Agree
- Neither agree nor disagree
- Disagree

1. The notifications/alerts were organized in a way that was easy to navigate (i.e., grouping by feature)

- No
- Yes

*Display This Question:*

*If The notifications/alerts were organized in a way that was easy to navigate (i.e., grouping by fea... = No*

1. What are some areas of improvement?

**The following 5 questions are specific to the COVID risk feature**

1. How easy is it to understand the data visualizations (e.g., graphs, charts)?

- Easy
- Neither easy nor difficult
- Difficult

*Display This Question:*

*If How easy is it to understand the data visualizations (e.g., graphs, charts)? = Difficult*

1. Please describe any issues you experienced
2. The level of control I have over visualizations (i.e., overall, by age, or day) helps me to understand the information

- Yes
- Indifferent
- No

1. I like the way the data visualizations appear (e.g., colours, size, type of graph)

- Yes
- Indifferent
- No

*Display This Question:*

*If I like the way the data visualizations appear (e.g., colours, size, type of graph) = No*

1. What are some ways that we could improve the appearance of the visualizations?
2. Based on the way the data was presented, I feel confident in my ability to use this data to make informed decisions regarding the community’s response to COVID (policy, announcement, etc.)

- Agree
- Somewhat agree
- Neither agree nor disagree
- Somewhat disagree
- Disagree

*Display This Question:*

*If Based on the way the data was presented, I feel confident in my ability to use this data to make... = Somewhat disagree*

*And Based on the way the data was presented, I feel confident in my ability to use this data to make... = Disagree*

1. What could have helped you feel more confident?
2. How long (in minutes) on average did it take you to respond to individual incidents?
3. Did you feel that this was too long?

**The following 3 questions are specific to the food security feature**

1. How easy is it to understand the data visualizations?

- Easy
- Neither easy nor difficult
- Difficult

1. The level of control I have over visualizations (i.e., overall, by age, or day) helps me to understand the information

- Yes
- Indifferent
- No

1. I like the way the data visualizations appear (e.g., colours, size, type of graph)

- Yes
- Indifferent
- No

*Display This Question:*

*If I like the way the data visualizations appear (e.g., colours, size, type of graph) = No*

1. What are some ways that we could improve the appearance of the visualizations?
2. Based on the way the data was presented, I feel confident in my ability to make informed decisions regarding an individual/household’s food security situation

- Agree
- Somewhat agree
- Neither agree nor disagree
- Somewhat disagree
- Disagree

*Display This Question:*

*If Based on the way the data was presented, I feel confident in my ability to make informed decision... = Somewhat disagree*

*And Based on the way the data was presented, I feel confident in my ability to make informed decision... = Disagree*

1. What could have helped you feel more confident?
2. How long (in minutes) on average did it take you to respond to individual incidents?
3. Did you feel that this was too long?

**The following 3 questions are specific to the citizen reporter feature**

1. How easy is it to understand the data visualizations?

- Easy
- Neither easy nor difficult
- Difficult

1. The level of control I have over visualizations (i.e., overall, by age, or day) helps me to understand the information

- Yes
- Indifferent
- No

1. I like the way the data visualizations appear (e.g., colours, size, type of graph)

- Yes
- Indifferent
- No

*Display This Question:*

*If I like the way the data visualizations appear (e.g., colours, size, type of graph) = No*

1. What are some ways that we could improve the appearance of the visualizations?
2. Based on the way the data was presented, I feel confident in my ability to make informed decisions regarding an individual/household’s incident?

- Agree
- Somewhat agree
- Neither agree nor disagree
- Somewhat disagree
- Disagree

*Display This Question:*

*If Based on the way the data was presented, I feel confident in my ability to make informed decision... = Somewhat disagree*

*And Based on the way the data was presented, I feel confident in my ability to make informed decision... = Disagree*

1. What could have helped you feel more confident?
2. How long (in minutes) on average did it take you to respond to individual incidents?
3. Did you feel that this was too long?

**General**

1. Is there any other feedback that you would like to share about your experience using the app?
