# Supplementary material for: Application of an Innovative Methodology to Build Infrastructure for Digital Transformation of Health Systems: Developmental Program Evaluation
Source: JMIR Form Res. 2025 Apr 17;9:e53339. doi: 10.2196/53339 (PMC12046263; doi:10.2196/53339)
Supplement: Multimedia Appendix 4 [file formative_v9i1e53339_app4.docx]

**Appendix D**

**Supplementary Table 1** Results from the post-pilot survey completed by community member participants during the pilot-test

| **Survey Question** | **Responses** | | | | | | | | | |
| --- | --- | --- | --- | --- | --- | --- | --- | --- | --- | --- |
| **User Experience** | **N=2 (100%)** | | | | | | | | | |
| 1. How well do you feel that the information used to create your avatar accurately reflects your risk of contracting COVID-19? | **Not accurately at all** | | | **Somewhat accurately** | | | | **Accurately** | | |
|  | 0 | | | 2 | | | | 4 | | |
| 2. After registering for the app... | **Agree** | | **Somewhat agree** | | | **Somewhat disagree** | | | **Disagree** | |
| a. I feel that the consent process was clear. | 6 | | 0 | | | 0 | | | 0 | |
| b. I feel that the app was easy to navigate. | 3 | | 3 | | | 0 | | | 0 | |
| c. I feel that I could find each app feature when needed. | 5 | | 1 | | | 0 | | | 0 | |
| d. I feel that my identity is anonymous. | 4 | | 2 | | | 0 | | | 0 | |
| e. I feel that I know who to contact if I have any questions for using the app or regarding my data or rights. | 5 | | 1 | | | 0 | | | 0 | |
| 3. Please describe any issues you experienced or suggestions for improvement: | N/A | | | | | | | | | |
| 4. How many notifications would you prefer to receive from the app? | **2-3 times/ day** | **Once/ day** | | | **2-3 times/ week** | | **Once/ week** | | | **It depends. Please specify:** |
|  | 0 | 5 | | | 1 | | 0 | | | 0 |
| 5. How long (in minutes) did it take you to complete your household avatars? | **≤5 minutes** | | | **5 minutes** | | | | **<10 minutes** | | |
|  | 1 | | | 3 | | | | 1 | | |
| 6. How many people did you create an avatar for? | **One person** | | | | | **Two people** | | | | |
|  | 4 | | | | | 2 | | | | |
| 7. Did you feel that this process was too long? | **No** | | | | | **Yes** | | | | |
|  | 5 | | | | | 1 | | | | |
| **COVID-19 feature** | **N=6 (100%)** | | | | | | | | | |
| 1. How easy did you find this feature to use? | **Easy** | | | **Neither easy nor difficult** | | | | **Difficult** | | |
|  | 5 | | | 1 | | | | 0 | | |
| 2. Please describe any issues you experienced or suggestions for improvement: | N/A | | | | | | | | | |
| 3. How comfortable were you interacting (e.g., reporting your social encounters) with this feature? | **Comfortable** | | | **Neither comfortable nor uncomfortable** | | | | **Uncomfortable** | | |
|  | 4 | | | 2 | | | | 0 | | |
| 4. Recommendations for my COVID risk were clear and easy to understand | **No** | | | | | **Yes** | | | | |
|  | 0 | | | | | 6 | | | | |
| 5. Please describe any issues you experienced | N/A | | | | | | | | | |
| 6. Which elements of this feature could be improved, and how? | “I had difficulty sending completed citizen reports, though the rest of the report features worked” “Having the link or app readily accessible through each reminder would have made it easier to find and ultimately use” “Allowing the device/search engine/app to save your login would have helped with accessibility (not sure if it's something you can control from your end, but I didn't get that option)” “Sometimes when you're on the web app it randomly goes back to the login screen, it may be a bug on the IOS Safari browser.” “I found it clear and easy to use. Maybe, there could be ability to add more details.” “It would be nice to see if my data was put in. I didn’t know if the information I entered went through or not” “The only confusing feature for me was creating a report because there was the arrow and the plus sign so I would press on the plus sign multiple times before noticing the arrow because I was using my phone and I would have to scroll down to see the arrow.” | | | | | | | | | |
| 7. How comfortable were you sharing your vaccination status? | **Comfortable** | | | **Neither comfortable nor uncomfortable** | | | | **Uncomfortable** | | |
|  | 5 | | | 1 | | | | 0 | | |
| **Food security feature** | **N=5 (83%)** | | | | | | | | | |
| 1. How easy did you find this feature to use? | **Easy** | | | **Neither easy nor difficult** | | | | **Difficult** | | |
|  | 5 | | | 0 | | | | 0 | | |
| 2. Please describe any issues you experienced | N/A | | | | | | | | | |
| 3. How comfortable were you using this feature (e.g., taking a photo of your fridge)? | **Comfortable** | | | **Neither comfortable nor uncomfortable** | | | | **Uncomfortable** | | |
|  | 3 | | | 2 | | | | 0 | | |
| 4. Which elements of this feature could be improved, and how? | “I can't comment on the bottom two questions as my food request was not responded to, still looks like it's on "sent".” “I felt the feature was set up well. Maybe the ability for users to provide more detail or select specific requests for food security.” “I didn’t know what kind of photo it was asking for. Maybe I missed it, but some instructions would be nice” “When I would send a question regarding my food order status, I did not get a response. I am not sure if it went through to whom ever would be responsible for that.” | | | | | | | | | |
| 5. How appropriate/sensitive was the response on the decision-makers end? | **Approp- riate** | **Some- what approp-riate** | | | **Neither approp-riate nor inappr-opriate** | | **Some- what inappro-priate** | | | **Inappro-priate** |
|  | 2 | 0 | | | 3 | | 0 | | | 0 |
| 6. How comfortable were you revealing your identity when support was needed? | **Comfortable** | | | **Neither comfortable nor uncomfortable** | | | | **Uncomfortable** | | |
|  | 3 | | | 2 | | | | 0 | | |
| **Citizen reporter feature** | **N=3 (50%)** | | | | | | | | | |
| 1. How easy did you find this feature to use? | **Easy** | | | **Neither easy nor difficult** | | | | **Difficult** | | |
|  | 2 | | | 0 | | | | 1 | | |
| 2. Please describe any issues you experienced | “I had attempted to send a citizen report since the first couple of days of this evaluation and it always took me through the process, but would never actually send in the end. Yunus suggested a few ways to solve it, but it did not work for me, so still unsure what the issue is.” | | | | | | | | | |
| 3. How comfortable were you interacting (e.g., reporting your incident, uploading photos) with this feature? | **Comfortable** | | | **Neither comfortable nor uncomfortable** | | | | **Uncomfortable** | | |
|  | 3 | | | 0 | | | | 0 | | |
| 4. Which elements of this feature could be improved, and how? | “I would not get a response so I am not sure if the report would go through or not” | | | | | | | | | |
| 5. How appropriate/sensitive was the response on the decision-makers end? | **Approp- riate** | **Some- what approp-riate** | | | **Neither approp-riate nor inappr-opriate** | | **Some- what inappro-priate** | | | **Inappro-priate** |
|  | 1 | 0 | | | 2 | | 0 | | | 0 |
| 6. How comfortable were you revealing your identity when support was needed? | **Comfortable** | | | **Neither comfortable nor uncomfortable** | | | | **Uncomfortable** | | |
|  | 2 | | | 1 | | | | 0 | | |
| 7. Are there ways we could improve this feature? | N/A | | | | | | | | | |
| 8. Is there any other feedback that you would like to share about your experience using the app? | “It's useful and provide clear information.” “I think the app could benefit from a tutorial on 'how to get started' to show the features of the app upon first login. I found it a bit difficult to find where to input my information at first.” “I found the app very straightforward, accessible, and organized. It was easy to navigate and find the features I wanted to use, and the data it provided regarding my risk was useful. Additionally, I found the tips the app provided relevant and helpful. One issue that I ran into was having to reset my password, as it wouldn't let me log in on few occasions. However, I was able to reset my password quickly and easily set a new one to access the app.” “Overall the app was great. However, it was hard to find on google and I using the app on the phone was sometimes difficult (i.e.. couldn’t see everything on my screen)” “It was easy to navigate and a good resource for a lot of information! It was easy to navigate and a good resource for a lot of information!” | | | | | | | | | |

**Supplementary Table 2** Results from the post-pilot survey completed by decision-maker participants during the pilot-test

| **Survey Question** | **Responses** | | | | | | | | | |
| --- | --- | --- | --- | --- | --- | --- | --- | --- | --- | --- |
| **User Experience** | **N=2 (100%)** | | | | | | | | | |
| 1. Do you feel that the information used to create your avatar accurately reflects your personal risk of contracting COVID-19? | **No** | | | | | **Yes** | | | | |
|  | **0** | | | | | 2 | | | | |
| 2. What pieces of information are missing? What else would you like to share about your risk? | N/A | | | | | | | | | |
| 3. After registering for the app… | **Agree** | | **Somewhat agree** | | | **Somewhat disagree** | | | **Disagree** | |
| a. I feel that the consent process was clear | 2 | | 0 | | | 0 | | | 0 | |
| b. I feel that the app was easy to navigate | 1 | | 1 | | | 0 | | | 0 | |
| c. I feel that I could find each app feature when needed | 1 | | 1 | | | 0 | | | 0 | |
| d. I feel that my identity is anonymous | 1 | | 1 | | | 0 | | | 0 | |
| e. I feel that I know who to contact if I have any questions for using the app or regarding my data or rights | 1 | | 1 | | | 0 | | | 0 | |
| 4. Please describe any issues you experienced | N/A | | | | | | | | | |
| **Decision-maker experience** | **N=2 (100%)** | | | | | | | | | |
| 1. I understand how to interact with a notification | **Agree** | | | **Neither agree nor disagree** | | | | **Disagree** | | |
|  | 2 | | | 0 | | | | 0 | | |
| 2. The notifications/alerts were organized in a way that was easy to navigate (i.e., grouping by feature) | **No** | | | | | **Yes** | | | | |
|  | 2 | | | | | 0 | | | | |
| 3. What are some areas of improvement? | N/A | | | | | | | | | |
| 4. Is there any other feedback that you would like to share about your experience using the app? | "It was great, but I feel that users will benefit from a presentation or a short video that could serve as reference material for later dates." "The FS part of the platform was easy to navigate and generally straightforward. It is an amazing amount of work to have simplified for the user. I wasn't sure if I could delete requests or if that is something only the mayor/tech department can do. Also, I wasn't sure if "citizens" could reach out to FS for questions or concerns about their food order (I tried that as a citizen but didn't receive a notification as a FS)." | | | | | | | | | |
| **COVID-19 feature** | **N=2 (100%)** | | | | | | | | | |
| 1. How easy is it to understand the data visualizations (e.g., graphs, charts)? | **Easy** | | | **Neither easy nor difficult** | | | | **Difficult** | | |
|  | 2 | | | 0 | | | | 0 | | |
| 2. Please describe any issues you experienced | N/A | | | | | | | | | |
|  | **Yes** | | | **Indifferent** | | | | **No** | | |
| 3. The level of control I have over visualizations (i.e., overall, by age, or day) helps me to understand the information | 2 | | | 0 | | | | 0 | | |
| 4. I like the way the data visualizations appear (e.g., colours, size, type of graph) | 2 | | | 0 | | | | 0 | | |
| 5. What are some ways that we could improve the appearance of the visualizations? | N/A | | | | | | | | | |
| 6. Based on the way the data was presented, I feel confident in my ability to use this data to make informed decisions regarding the community’s response to COVID (policy, announcement, etc.) | **Agree** | **Somewhat agree** | | | **Neither agree nor disagree** | | **Somewhat disagree** | | | **Disagree** |
|  | 1 | 1 | | | 0 | | 0 | | | 0 |
| 7. What could have helped you feel more confident? | N/A | | | | | | | | | |
| 8. How long (in minutes) on average did it take you to respond to individual incidents? | **2 minutes** | | | | | **Less than 5 minutes** | | | | |
|  | 1 | | | | | 1 | | | | |
| 9. Did you feel that this was too long? | **Yes** | | | | | **No** | | | | |
|  | 0 | | | | | 2 | | | | |
| **Food security feature** | **N=2 (100%)** | | | | | | | | | |
| 1. How easy is it to understand the data visualizations? | **Easy** | | | **Neither easy nor difficult** | | | | **Difficult** | | |
|  | 2 | | | 0 | | | | 0 | | |
|  | **Yes** | | | **Indifferent** | | | | **No** | | |
| 2. The level of control I have over visualizations (i.e., overall, by age, or day) helps me to understand the information | 2 | | | 0 | | | | 0 | | |
| 3. I like the way the data visualizations appear (e.g., colours, size, type of graph) | 2 | | | 0 | | | | 0 | | |
| 4. What are some ways that we could improve the appearance of the visualizations? | N/A | | | | | | | | | |
| 5. Based on the way the data was presented, I feel confident in my ability to make informed decisions regarding an individual/household’s food security situation | **Agree** | **Somewhat agree** | | | **Neither agree nor disagree** | | **Somewhat disagree** | | | **Disagree** |
|  | 2 | 0 | | | 0 | | 0 | | | 0 |
| 6. What could have helped you feel more confident? | N/A | | | | | | | | | |
| 7. How long (in minutes) on average did it take you to respond to individual incidents? | **2 minutes** | | | | | **<5 minutes** | | | | |
|  | 1 | | | | | 1 | | | | |
| 8. Did you feel that this was too long? | **Yes** | | | | | **No** | | | | |
|  | 0 | | | | | 2 | | | | |
| **Citizen reporter feature** | **N=2 (100%)** | | | | | | | | | |
|  | **Easy** | | | **Neither easy nor difficult** | | | | **Difficult** | | |
| 1. How easy is it to understand the data visualizations? | 2 | | | 0 | | | | 0 | | |
|  | **Yes** | | | **Indifferent** | | | | **No** | | |
| 2. The level of control I have over visualizations (i.e., overall, by age, or day) helps me to understand the information | 2 | | | 0 | | | | 0 | | |
| 3. I like the way the data visualizations appear (e.g., colours, size, type of graph) | 2 | | | 0 | | | | 0 | | |
| 4. What are some ways that we could improve the appearance of the visualizations? | N/A | | | | | | | | | |
| 5. Based on the way the data was presented, I feel confident in my ability to make informed decisions regarding an individual/ household’s incident? | **Agree** | **Somewhat agree** | | | **Neither agree nor disagree** | | **Somewhat disagree** | | | **Disagree** |
|  | 2 | 0 | | | 0 | | 0 | | | 0 |
| 6. What could have helped you feel more confident? | N/A | | | | | | | | | |
| 7. How long (in minutes) on average did it take you to respond to individual incidents? | **2 minutes** | | | | | **Less than 5 minutes** | | | | |
|  | 0 | | | | | 1 | | | | |
| 8. Did you feel that this was too long? | **Yes** | | | | | **No** | | | | |
|  | 0 | | | | | 1 | | | | |
